# Modelling Pedestrian Travel Time and the Design of Facilities: A Queuing Approach

**DOI:** 10.1371/journal.pone.0063503

**Published:** 2013-05-15

**Authors:** Khalidur Rahman, Noraida Abdul Ghani, Anton Abdulbasah Kamil, Adli Mustafa, Md. Ahmed Kabir Chowdhury

**Affiliations:** 1 Mathematics Section, School of Distance Education, Universiti Sains Malaysia, Penang, Malaysia; 2 School of Mathematical Sciences, Universiti Sains Malaysia, Penang, Malaysia; 3 Department of Statistics, School of Physical Sciences, Shahjalal University of Science and Technology, Sylhet, Bangladesh; National Research & Technology Council, Argentina

## Abstract

Pedestrian movements are the consequence of several complex and stochastic facts. The modelling of pedestrian movements and the ability to predict the travel time are useful for evaluating the performance of a pedestrian facility. However, only a few studies can be found that incorporate the design of the facility, local pedestrian body dimensions, the delay experienced by the pedestrians, and level of service to the pedestrian movements. In this paper, a queuing based analytical model is developed as a function of relevant determinants and functional factors to predict the travel time on pedestrian facilities. The model can be used to assess the overall serving rate or performance of a facility layout and correlate it to the level of service that is possible to provide the pedestrians. It has also the ability to provide a clear suggestion on the designing and sizing of pedestrian facilities. The model is empirically validated and is found to be a robust tool to understand how well a particular walking facility makes possible comfort and convenient pedestrian movements. The sensitivity analysis is also performed to see the impact of some crucial parameters of the developed model on the performance of pedestrian facilities.

## Introduction

Excessive environmental pollution, shrinking energy resources, and mounting awareness of “back-to-nature” for physical fitness have accentuated walking as a popular transportation mode to the urban dwellers. Therefore, there has been a necessity to design and construct the urban pedestrian facilities based on the well-recognized local parameters and flow characteristics [1], [2]. On the other hand, the design and layout of walking facilities also have considerable impact on the comfort and expedient movements of pedestrian. Thus, the design and layout should be in favour of pedestrian movements and optimize the performance or the serving capacity of a facility. There are different methods for assessing the performance of different walking facilities. Some performance measures reflect operating conditions such as traffic, and control conditions of a facility. Mitchell & MacGregor Smith [3] considered pedestrian throughput through the facility, the average number of pedestrians within the facility at steady-state, and the probability of balking as performance measures of a pedestrian facility. Other studies [4], [5], [6] have suggested that occupant flow density (OFD), freedom to manoeuvre, and the delay, comfort and convenience experienced by the pedestrians could be used as performance measures. Each performance measure that is a level of service (LOS)-determining parameter in pedestrian walkways is called the service measure or sometimes the measure of effectiveness (MOE) for a particular facility.

LOS is related to the qualitative evaluation of traffic flow characteristics. The roadway traffic engineers first introduced the concept of LOS based on the philosophy that capacity design, the absolute maximum attainable vehicular flow rate, is not required on all roadways. As such designing could rather deteriorate into intermittent go and stop movement of vehicles. Analogous to the LOS descriptions for vehicular flow [7], based on the analysis of body dimensions and observations of queues, Fruin [8] defined six LOS standards for pedestrian locomotion in the roadside walking environment. In fact, his definition of LOS standards is based on the free expanse area that could be available for a single pedestrian. A similar definition is provided by Oeding [9], Pushkarev and Zupan [10] for the LOS. But their defining ranges of area for a single pedestrian are different from those of Fruin’s. Seneviratne and Morrall [11], however, defined the concept of LOS in actual relation to people’s behavior and perceptions. In addition, calibrating and evaluating three-regime linear speed-density regression model, Polus, Schofer & Ushpiz [12] proposed four types of LOS in terms of pedestrian densities. They also provided suggestions for using their defined LOS concepts in the planning and design of different types of walk paths. The maximum rate of pedestrian volumes that could be flowed on a particular facility at each LOS was also estimated. However, a clear suggestion on the sizing of pedestrian walkways was not provided.

Pedestrian movements are the consequence of several complex and stochastic facts. Recognizing the manner of pedestrian movements is very helpful to the traffic engineers who are responsible for providing acceptable LOS, capacity analysing and designing the pedestrian facilities. In addition, pedestrian travel time is one of the important characteristics in the field of transportation, which can be used as a good descriptor of the LOS offered by a walkway [11], [13]. Thus, the modelling of the pedestrian movements and ability to predict the travel time are useful to investigate and improve the pedestrians’ perception of the capability of walking facilities. These also help in terms of minimizing pedestrian conflicts and expenditure of human energy. Although the pedestrian movements are influenced by several factors, a number of studies have found that the useful tools for designing and improving pedestrian facilities could be developed based on the fundamental relationship of speed-density i.e., the travel time and density relationship [1], [2]. A variety of linear and non-linear models has been developed for travel time/speed and density relationship. However, no model has been developed, that, in addition to density, incorporate the layout of the facility, local pedestrian body dimensions, and the delay experienced by the pedestrians to the pedestrian travel time. Development of a proper single-regime model will be helpful in determining the size of a facility required to provide a predetermined LOS [11].

Therefore, the main goal of the current study is to develop an accurate analytical model for pedestrian travel time on walkways/pedestrian facilities that can recognize the stochastic nature of the pedestrian flows and incorporate the relevant information. The model will also be used to assess performance, planning and designing of pedestrian facilities. Thus, the developed model, based on queuing theory, will be helpful to traffic engineers to understand how well a particular sidewalk makes possible comfort and convenient pedestrian movements.

The remainder of this paper is described in the following manner. The background literature related to the research on pedestrian travel time and the research contributions of the current study are provided in the next section. Then the relevant determinants and functional factors that should be considered in the modelling of pedestrian travel time and the design and capacity analysis of walkways/pedestrian facilities are discussed. After that, the proposed model is formulated and described as a function of relevant determinants and functional factors. In the section following the formulation, the use of queuing theory for modelling pedestrian travel time is empirically validated. Little attention has been devoted to study the design parameters for pedestrian facilities in developing countries. Thus, the pedestrian flow characteristics on a sidewalk in Dhaka, Bangladesh, a typical capital city of least developing countries, have been considered as a case study to verify the accuracy of the developed model. The sensitivity analysis is also performed to see the impact of some crucial design and pedestrian ability parameters of the developed model on the performance and capacity of pedestrian facilities. Finally, conclusions, limitations, and the recommendations for extensions of the current research are provided.

### Background Studies and Benefits of the Current Study

Over the last five decades, the well-known Bureau of Public Roads (BPR) [14] model has been broadly used by traffic researchers and policy makers for estimating travel times on road networks. This model has also been adopted, in the following form, in estimating pedestrian travel time on different walking facilities:
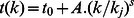
(1)where:

*t(k)* = travel time (sec ) at flow level or density *k;*

*t_0_* =  free-flow travel time (sec);

*k* =  pedestrian flow (peds/m/sec) or density (peds/m^2^);

*A, s* = constants to be estimated in the model fitting procedure;

*k*_j_ =  the capacity of the pedestrian facility (peds/m/sec or peds/m^2^) and


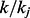
 =  pedestrian demand to capacity ratio.

The practical measurement of travel time considers the queuing time as well as the time required for a pedestrian to travel along the facility [15]. The formula in Eq.(1) is only applicable when the value of *k* is less than the value of *k*_j_.

The pedestrian simulation-assignment model, PEDROUTE [16], used Eq.(1) to estimate pedestrian travel times in the congested London Underground (LU) system. Following this, several studies [17], [18], [19], [20] have adopted the equation to predict pedestrian travel times and to establish the pedestrian speed/flow relationships. However, Al-Masaeid, et al. [21] used the pedestrian demand to capacity ratio to study the percentage of pedestrians walking along the street beside a sidewalk. In addition, adapting the test vehicle technique [22], Virkler [13] developed a new method that used the test pedestrians to predict and measure the walking and queuing times on pedestrian routes.

Although the waiting time or delay experienced by a pedestrian on a walking facility was considered in empirical measurement in the previous studies based on Eq.(1), however it was not explicitly reflected in the formulation of the model for travel time. The models and measurements also did not explicitly take into consideration the local pedestrian body dimensions (e.g. the lateral spacing for movements) and walking capacity (e.g. free flow speed). In addition, these models have limited ability to provide a platform for sensitivity analysis. The current queuing theory based model, although an abstract of reality, overcomes these problems and can formulate the complex and stochastic pedestrian movements and travel time in a comprehensive way. The model is specifically formulated for sidewalks and can be used to assess the influence of relevant determinants and functional factors, which are discussed in the next section on the basic determinants and functional factors.

The developed model assumes steady-state equilibrium conditions, i.e. the same probabilities are produced by the repeated observations of same pattern of flows. Such assumption is necessary for recommending appropriate design and policy. The study presented here is macroscopic meaning that the study considered the movements of all pedestrians in a pedestrian facility and aggregates their characteristics to uninterrupted bidirectional pedestrian flow [23]. Thus, the study is a new endeavour to provide the traffic engineers with the guidelines for space allocation for pedestrians (the width and length of the facility) with an acceptable level of congestion, ensuing in an obvious suggestion on the sizing of sidewalks at the Central Business Districts (CBDs). The developed model is more appropriate for the study of sidewalks which are connected by over-bridges and flows are not interrupted crosswalk signals or others. Furthermore, with some modifications, the model can be used in the design and analysis of a multi-line highway. Thus, the developed model can be used to assess the multi-modal LOS for transit, pedestrian, and bicycle modes [24].

### The Basic Determinants and Functional Factors

A superior design for pedestrian facilities is the one which is based on a model that can incorporate all the relevant determinants and functional factors. Among a number of determinants and functional factors [1], [2], [25], the most important ones, which are included in the current developed model, are listed below. In addition to civil engineering concepts and the traffic engineer’s idea, these determinants and functional factors should be adhered to the modelling of travel time and the designing of pedestrian facilities.

### Free Flow Speed

The speed by which a pedestrian desires to walk when she/he is not hindered by other nearby pedestrians is defined as free flow speed [26]. It is usually denoted by *v_f_.* This quantity plays a significant role in developing models that are relevant in the design and improvement of pedestrian facilities, and public transport timetables. In addition, the determination of free flow speed is necessary to evaluate the constraints on pedestrian movements that occur at different level of concentrations (densities) of traffic [8]. The free flow speed is likely to be more sensitive to personal characteristics (age, gender, baggage carrying capacity of a pedestrian), type of walking facility (grade, indoor or outdoor pedestrian facility), and ambient factors (walkability, weather). This speed also differs among the pedestrians from different countries and regions of the world [1]. Thus, the value of local pedestrian free flow speed should be incorporated in the modelling of travel time and designing of pedestrian facilities. The inclusion of free flow speed partially associates the stochastic nature of the pedestrian movements.

### Maximum/Jam Density

The jam density refers to the maximum admissible density corresponding to null speed and flow [27]. It is usually estimated by fitting a speed-density curve and denoted by *k_j_*. As the pedestrian traffic density increases, the movement of faster pedestrians is likely to be impeded by slower pedestrians. At a certain density, walking is reduced to a shuffle, and at jam density, forward movement is halted [28]. Like free flow speed, jam density differs among the pedestrians from different countries and regions of the world [2]. This is due to the differences in pedestrians’ body dimensions and walking capacities. Thus, the quantitative value of jam density that is estimated on the basis of local pedestrians’ flow characteristics should be incorporated in the modelling of travel time and designing of pedestrian facilities. However, this study found that the observed maximum density (capacity of the facility) is more useful to the developed model than the estimated jam density. Such observed maximum densities were also employed in the use of the BPR model for pedestrian travel time [15], [18]. The observed maximum density, at which pedestrians can move, is always less than the jam density. Thus, observed maximum density is more suitable to be used for the modelling of pedestrian movements as well as for travel time on the sidewalks.

### Effective width of the Facility

In designing a pedestrian facility (e.g. sidewalks), the traffic or civil engineers should pay special attention to the length and width of the facility. After the determination of basic layout, usually the length of a walking facility is determined based on the function and purpose of the facility. When the length is set, it is necessary to determine the width of the facility. The effective width of a sidewalk facility, which generally remained constant throughout the facility, should be determined to provide the comfort and convenient movements to the pedestrians.

In a flow of mass pedestrians, pedestrians are forced into an orderly pattern of movements – the flow behaves similar to that of vehicles on highway, and pedestrians often have to walk in lines of more or less straight lines [11]. Thus, the lane concept as like the highway design can be used in pedestrian flow to estimate how many pedestrians can walk abreast or pass each other simultaneously in a facility with fixed width. When two persons pass each other in a bidirectional flow on the walkway, the required lateral spacing/width for each pedestrian to avoid interference is from 0.75 m to 0.80 m [4], [10], [29]. However, this range is from 0.65 m to 0.70 m, if two pedestrians are acquainted with each other [4], [10]. In analysing a pedestrian facility the unused space on the edges, due to the curb and walls, should be reduced from the available width. According to Navin & Wheeler [29], 1.07 m of width should be reduced from the available width to calculate the effective width of a sidewalk. Thus, the theoretical capacity of a walkway part will be greater than the actual capacity value. Furthermore, the fundamental equation for pedestrian traffic flow can be used for sizing the width of a facility [30] and are given as:
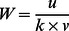
(2)where:

*W* = width of the facility;

*u* = volume of pedestrians (peds/sec.);

*v* = pedestrian mean/effective speed (m/sec.) and

*k* = pedestrian mean density or concentration (peds/m^2^).

Thus, if the pedestrian effective speed is known, the width of a walking facility can be estimated. In addition, as explained in first section and the subsequent sections, the speed and/or travel time of the pedestrians can be used as a good descriptor of the LOS offered by a walkway.

### Level of Service: Overall Service Rate

The overall service rate as defined below in Eq. (3), has a correlation with the LOS, that can be used to assess the performance of a pedestrian facility. This is because, in terms of pedestrian densities, there is an interaction between the LOS and the pedestrian speed/travel time [12]. This interaction may be used to determine the size of facility required to provide a predetermined LOS [11]. Thus, the pedestrian travel time is considered in the formulation of overall service rate and is given by [31]:
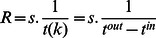
(3)where:

*R* = overall service rate of the facility;

*s* = the number of pedestrians that can be accommodated in the minimal length of the facility;

*t^in^* = time (sec.) of go in of a pedestrian in the facility;

*t^out^* = time(sec.) of go out of a pedestrian from the facility and

*t(k) = t^out^- t^in^* =  travel time(sec.) at density *k.*

Thus, the overall service rate or the performance of a facility, in terms of travel time, depends on the pedestrian ability to walk or free flow speed, prevailing density or LOS in the flow, lateral spacing required for a pedestrian, maximum admissible density or jam density on the facility, and the required waiting time or lingering time, respectively. The model in Eq. (3) will be formulated in the following section so that all these factors are incorporated.

### The Queuing Based Analytical Model

**Figure 1:**
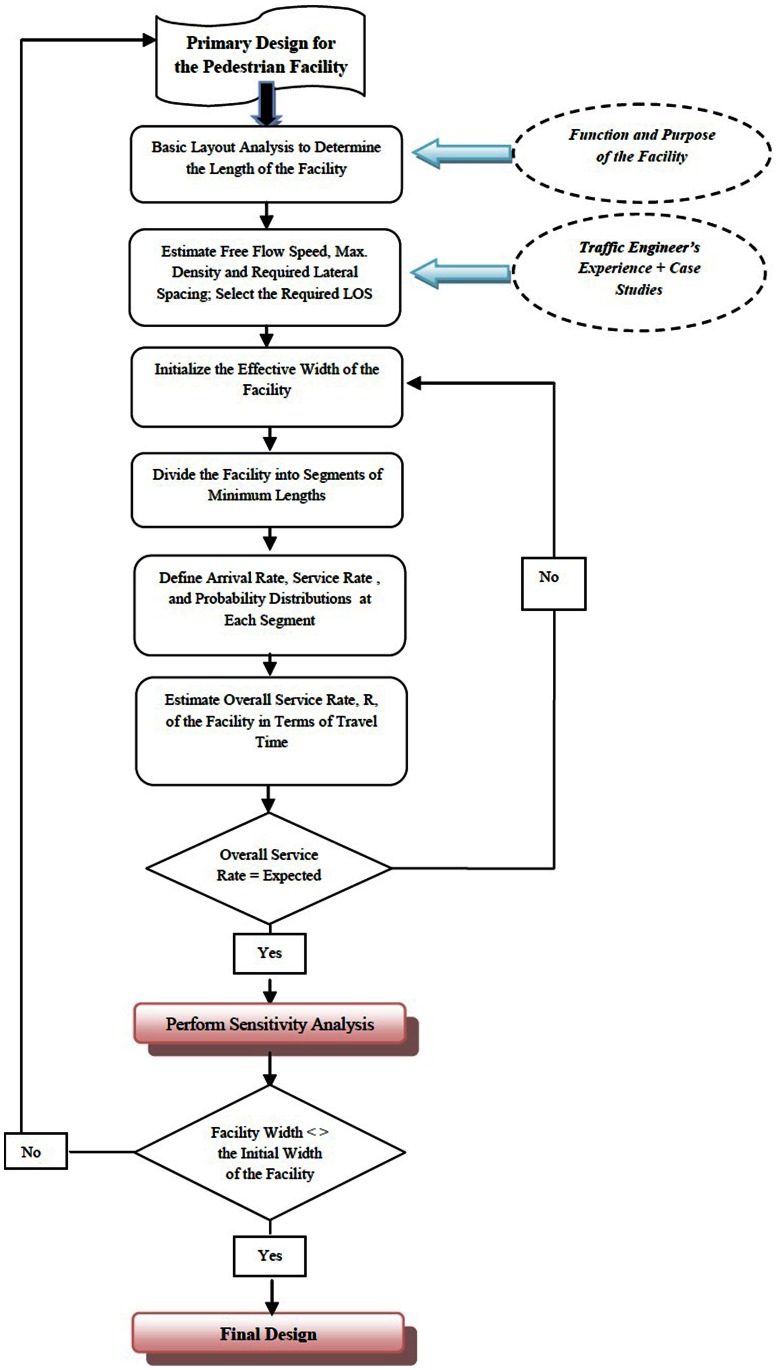
Process for Computing Overall Service Rate.

First, we will develop a travel time model based on the queuing theory. Since the overall service rate can be estimated by the required travel time, which is influenced by the prevailing LOS, we could utilize the developed model to assess the LOS for a given overall service rate. Moreover, the structure presented in Fig. 1 can be used to subject the queuing model in an integrated design environment for pedestrian facilities. The details of formulation and computation process are explained in the following.

The queuing theory, a branch of stochastic processes, has been prominently used as an analytical tool in the field of operation research since the beginning of 21st century (for details, see [32] ).The theory is based on the concept that a queue or waiting line is formed when the customers (e.g. pedestrians) need more service(s) on arrival at service node(s) than they are provided. Queuing theory studies these waiting lines through mathematical models. The basic entities which characterize a queuing model are: i) the arrival rate, ii) the service mechanism, iii) the queue discipline (e.g. *first come first served, FCFS*), and iv) the number of service nodes.

In this study, the concept of a pedestrian lane concept is used to model the pedestrian travel time as well as the performance of a facility based on queuing theory, similar to modelling a highway lane. The developed model is based on the following assumptions:

- The pedestrian facility (after the subtraction of the unused spaces) is virtually horizontally divided into lanes, where the width of a lane is the lateral spacing required for a pedestrian to move in the presence of other pedestrians. Each lane is then vertically further subdivided into a sequence of segments with equal length. The length of each segment is the minimal length required for a pedestrian standing room in the presence of others.
- The pedestrian facility acts as a service provider where a number of servers (lanes) are working in a station of sequential service stations (segments). In each service station (segment), pedestrians arrive at a certain rate 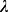 and get served at another rate 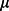.
- Pedestrians are uniformly distributed over the facility (lanes and segments) at each moment of time.

To gain a clear understanding of the above concept and assumptions we can use the following notations:

*L and W* = length (m) and width (m) of the facility, respectively;

*k and k_j_* = prevailing density (peds/m^2^) and maximum/jam density (peds/m^2^), respectively;

*n_i_ and n_j_* = number of pedestrians on the facility, (peds), at prevailing density and maximum/jam density, respectively;

*b* = lateral spacing required for a pedestrian to move (m);

*s* = 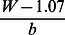 = number of servers in a station (segment);

*v_f_* = average free flow speed of pedestrians (m/sec);

*T, T_p_ and T_q_* = the expected total time (sec), the expected service time (sec) and the expected waiting time (sec) due to congestion, respectively, for a pedestrian to pass through a segment of the facility, and 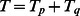.

Thus, following the use of queuing approach to vehicle flow [33], [34] the arrival rate 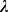 and the service rate 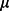 at each station (segment) can be defined as:
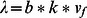
(4)and



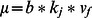
(5)Since units for *b, k, k_j_* and *v_f_* are m, peds./m^2^, peds/m^2^ and m/sec, respectively, both 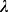 and 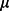 are rates in unit peds/sec. As the values of *b, k_j_* and *v_f_* are considered fixed for a particular region and pedestrian facility, in our formulation the arrival rate 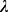 (depending on the value of *k*) could vary, but the service rate 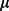 is fixed. This consideration holds opposing views to the analysis based on state dependent queuing models [3], [31].

In addition, the minimal length required for a pedestrian to move in the presence of others is 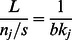. When the expected value of *T* is known, we can easily calculate the effective speed as [35]:
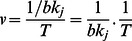
(6)


The expected total time, in Eq.(6), that a pedestrian spends on the facility to pass one of the segments, depends upon the queuing model considered to resemble the pedestrian flow. Here, we are concerned with the model which envisages a queuing system with *s* servers working in parallel at a station and having identical arrival and service time distributions. Thus, in Kendall notation, our queuing system is expressed by M/M/s. We assume that the queue discipline is FCFS.

As described in the above, we can consider that each segment (station) of the pedestrian facility has a finite capacity to facilitate the passing of *s* pedestrians at a time. In our consideration the pedestrians arrive in each segment in accordance with a Poisson process having an average rate 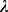. In the lateral movement, when 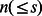 pedestrians can simultaneously pass a segment, the segment could provide services in Poisson process with mean 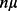 and the time between two pedestrians to pass the segment is exponential with mean 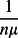; whereas when 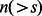 pedestrians want to pass a segment, the time between two pedestrians to pass the segment is exponential with mean 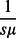. The steady-state solutions of the probabilities, *p_n_*,*n* = 1,2,……., of *n* pedestrians and 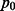 of no pedestrian in a segment are as shown in Eq. (7) and Eq. (8), respectively, for 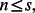


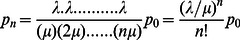
(7a)and, for 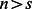


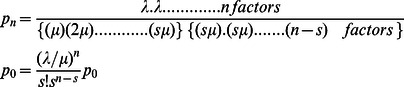
(7b)such that,
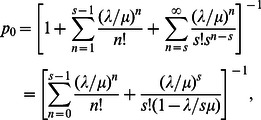
(8)since, from Eqs. (4) and (5), we will always have the condition 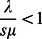.

Thus, using Eq. (7) and Eq. (8), it can be shown that the expected waiting time (i.e. the delay experienced by a pedestrian) and total time can be expressed as follows [32]:
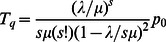
and



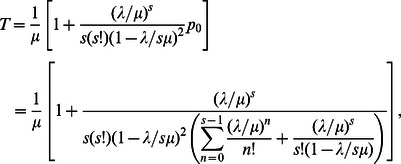
using Eq.(8)



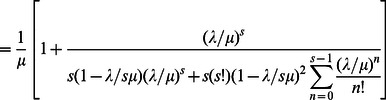
(9)Now, using Eqs.(4), (5) and (9) in Eq.(6), we get the formula for the effective speed as.
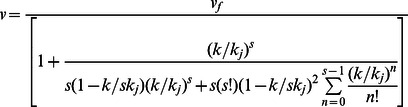
(10)


If we consider *s = 1* in Eq.(10) than the equation becomes as.
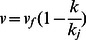
(11)


This is the well-known Greenshields model which has been used since 1935 in the study of the vehicle as well as the pedestrian speed-flow-density relationships [36], [37]. Thus, if we consider single pedestrian lane or if we consider that each segment serves as a single-server with the capacity to serve *s* pedestrians simultaneously, then our model becomes as M/M/1. However, in the later case, we have to consider the bulk arrival and the bulk service of size *s* in each segment, which is not realistic for the moving pedestrians on the sidewalks.

Again, using Eq.(10), the model for average travel time required for a pedestrian, on a facility with length L, becomes. 
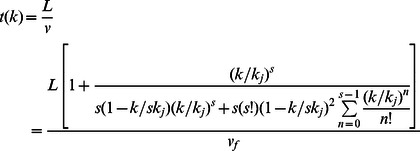
(12)


This equation explicitly includes factors that affect the pedestrian travel time compared to Eq.(1) i.e., includes walk or free flow speed, prevailing density or LOS in the pedestrian flow, lateral spacing required for a pedestrian, maximum admissible density or jam density on the facility, length and effective width of the facility, as well as experienced waiting time. The model in Eq. (12) has been tested against the real travel time data collected from a sidewalk of Dhaka, Bangladesh and close estimate of pedestrian travel time is found that will be described in the next section.

Note that *L/v_f_* = *t_0_* =  free-flow travel time. Thus, we can reformulate the above Eq.(12) as.
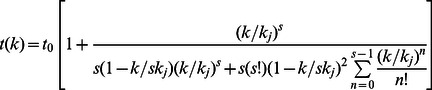
(13)


Hence, by superimposing Eq.(13) on Eq.(1) we have.
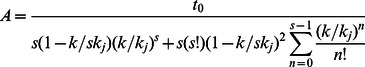
(14)


For the given values of *t_0_, k_j_* and *s*, this *A* is a function of *k* (prevailing LOS), whereas in Eq. (1) *A* is considered as a constant value. This difference is due to the waiting time or lingering time, which is included in the derivation of Eq. (13). The developed model considered that the percentage of the contribution of capacity ratio 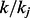 to travel time *t(k)* cannot be constant due to change in waiting time. Another difference is that an iterative procedure is required to find the *s* in Eq. (1) using the empirical data; whereas it has to take a meaningful value in Eq. (13). Since the value of *s* in Eq. (13) is determined based on the pedestrian required lateral spacing and effective width, the derived model can be used to correlate the LOS in the cost-effective and efficient sizing of pedestrian facilities.

To correlate the LOS to the performance of a facility in terms of pedestrian travel time we can use Eq.(12) in Eq.(3) to produce the following overall service rate model. 
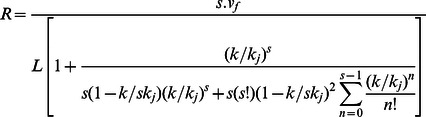
(15)


Using this equation, we can get a numerical solution for s, i.e. the necessary width for a given overall service rate and LOS. Equation (15) provides a more descriptive design model than Eq.(2) as it incorporates several additional design determinants and factors that have been mentioned earlier. The traffic engineer can utilize Eq. (15) to investigate and perform sensitivity analysis for pedestrian facilities that will be described after empirical validation.

### Empirical Validation

Data on the pedestrian average free flow speed and flow characteristics on some sidewalks in Dhaka, Bangladesh had been collected by Rahman, et al. [1], [2] and have been used to verify the accuracy of the developed model. Rahman, et al. [1] reported that the estimated average free flow speed for the pedestrians on sidewalks in Dhaka is 1.2 m/sec. Using weighted regression on the pedestrian flow characteristics of three sidewalks, Rahman, et al. [2] also found the estimated jam density for Dhaka sidewalks as 3.32 peds/m^2^. Among the selected sidewalks, the Commissioner’s Market Sidewalk, Farmgate (a busy place in Dhaka) had the wide range of densities. Thus the flow characteristics at this sidewalk have been selected to validate the developed model in Eq. (12). The flows on the Commissioner’s Market Sidewalk are uninterrupted and the sidewalk is connected with Kazi Nazrul Islam Avenue by two over-bridges. Data on pedestrian movements were recorded both peak and off-peak periods under clear and dry weather. To compare the pedestrian real travel time data with the estimated travel time data, a paired-samples *t*-test was conducted.

**Figure 2:**
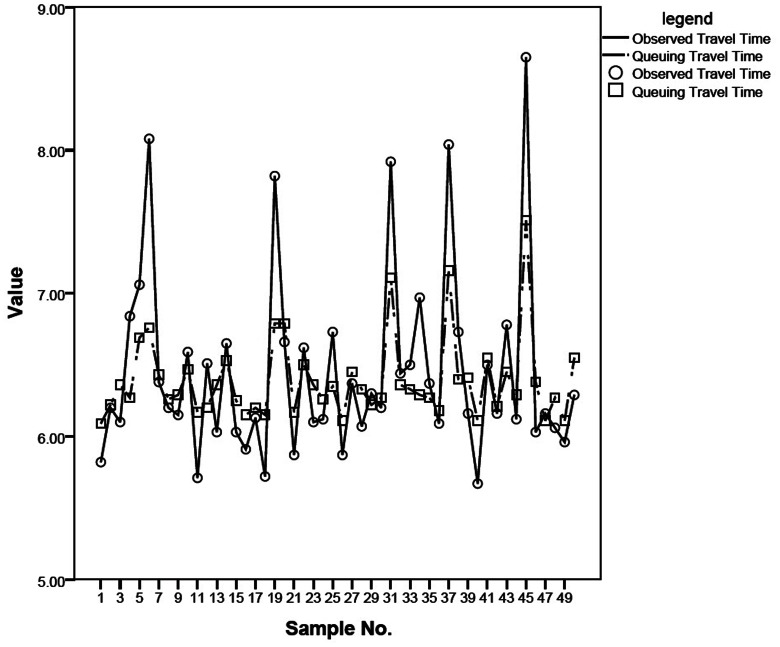
The queuing travel time vs the observed travel time.

At first attempt, the estimated quantities of free flow speed and jam density by Rahman, et al. [1], [2] and the lateral spacing 0.8 m as mentioned in Manual [4] were fed into the developed model to estimate the travel times for pedestrians. Statistically significant (at 5 percent level) differences were found between the averages of actual travel times and estimated travel times. However, instead of jam density, when the observed maximum density of 1.55 peds/m^2^ (as used in the previous studies based on Eq. (1)) and also the location-specific average free flow speed (1.10 m/sec.) was used, no statistically significant differences were found between the averages. The results of empirical validation are given in table 1 and shown in Fig. 2, which show that the M/M/s based developed model could precisely predict the pedestrian travel times on the selected sidewalk.

**Table 1:** A Comparison between the Actual and Estimated Pedestrian Travel Time.

Measured average pedestrian travel time (sec)	Estimated (sec)	Discrepancy	Accuracy	p-value (t-test)
6.45	6.38	0.06	99.06%	0.323

The unsuitability of using the jam density supports the remark of Seneviratne & Morrall [11] and Polus et. al [12]. They mentioned that due to the freedom of movements in walking, it is difficult to observe heavily congested conditions on sidewalks. Another point is that the jam density is estimated considering the condition when forward movement is halted, which is rare to be observed in a sidewalk condition. In addition, Fruin [8] suggests the density of 1.55 ped./m^2^ as a limit above which personal contact with others is unavoidable. It should be noted that pedestrians on sidewalks always try to avoid the close touch of others. Hence, the density 1.55 ped./m^2^ is a reasonable value to be considered as a maximum density. In addition, the suitability of location-specific average free flow speed confirms that the “walkability” of a facility has influence on pedestrian movements’ quality [38].

**Figure 3:**
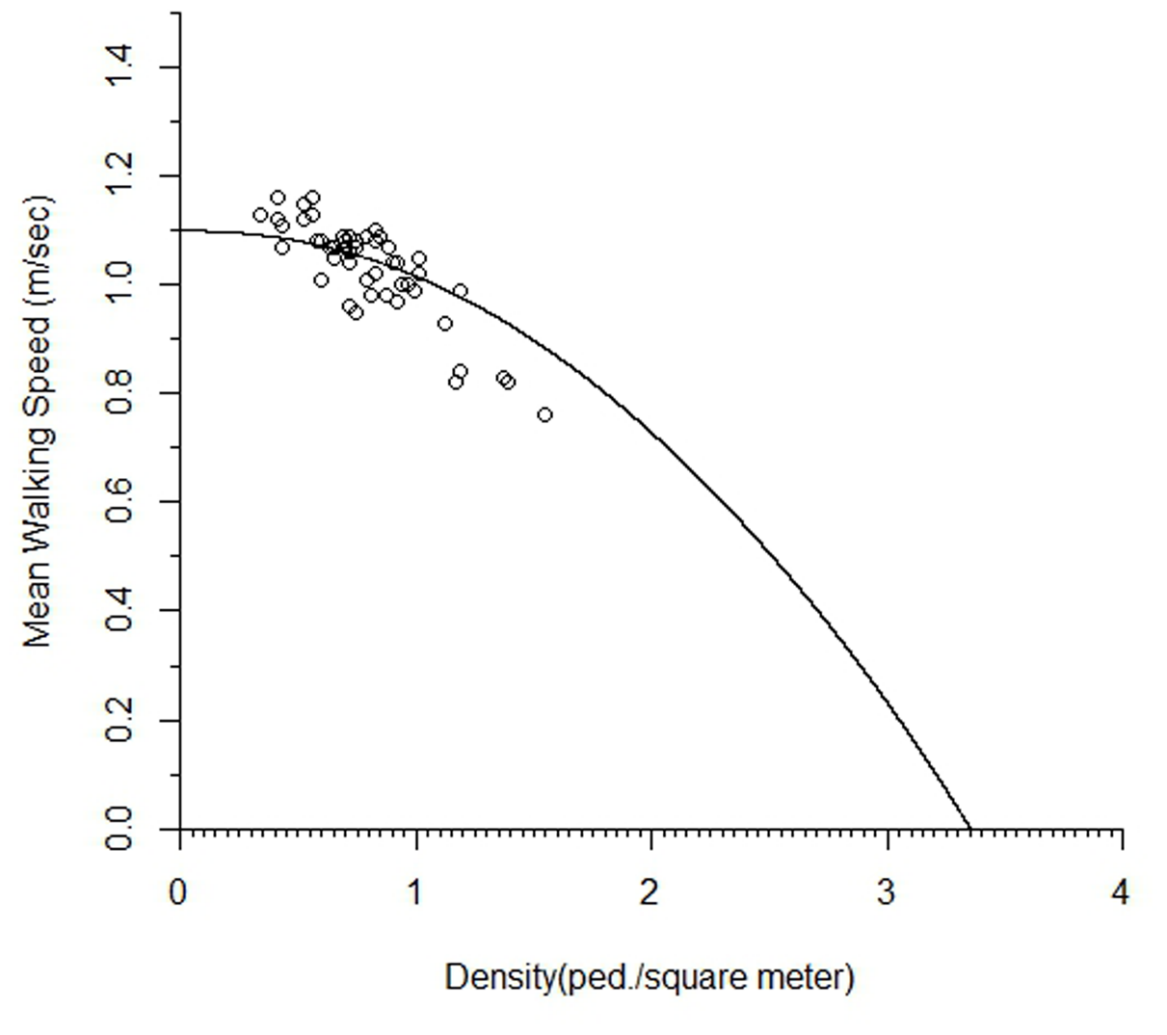
The speed-density diagram for the M/M/s queuing model.

Furthermore, Fig. 3 depicts the scatter diagram for the speed-density relationship in conjunction with the M/M/s queuing system based developed model in Eq. (10). The fitted curve shows that pedestrian movements of the selected location will be halted at the jam density of 3.35 ped/m^2^. This jam density is lower than those of uni-directional flows and emergency evacuation situations [10], [11], [28], [39], [40]. Navin and Wheeler [29] noted that the two- way flow reduces the number of paths and manoeuvring space. It also generates collisions between pedestrians, which in turn reduces total flow and produces lower value of jam density. Mauron [41] in his thesis, by simulating the pedestrian movements, observed that in high densities 5 percent of opposing traffic reduces the mean speed by 50 percent relative to uni-directional flow.

To facilitate the sensitivity analysis and applications for other sidewalks as well as for other types of facilities a computerized study was done using R statistical language and is described next. The required codes are also available in the Appendix S1.

### Sensitivity Analysis and the Design for Pedestrian Facilities

The impact of different parameters of the developed model on the overall service rate or performance can be investigated by a sensitivity analysis. For this purpose, the sidewalk selected for empirical validation was further analysed to predict the overall service rate and to see the utility of the developed model on the sizing and improvement of pedestrian facilities. The main variable, the prevailing density or LOS, and the most important parameter, the width of the sidewalk, were considered to observe the impact on the performance.

**Figure 4:**
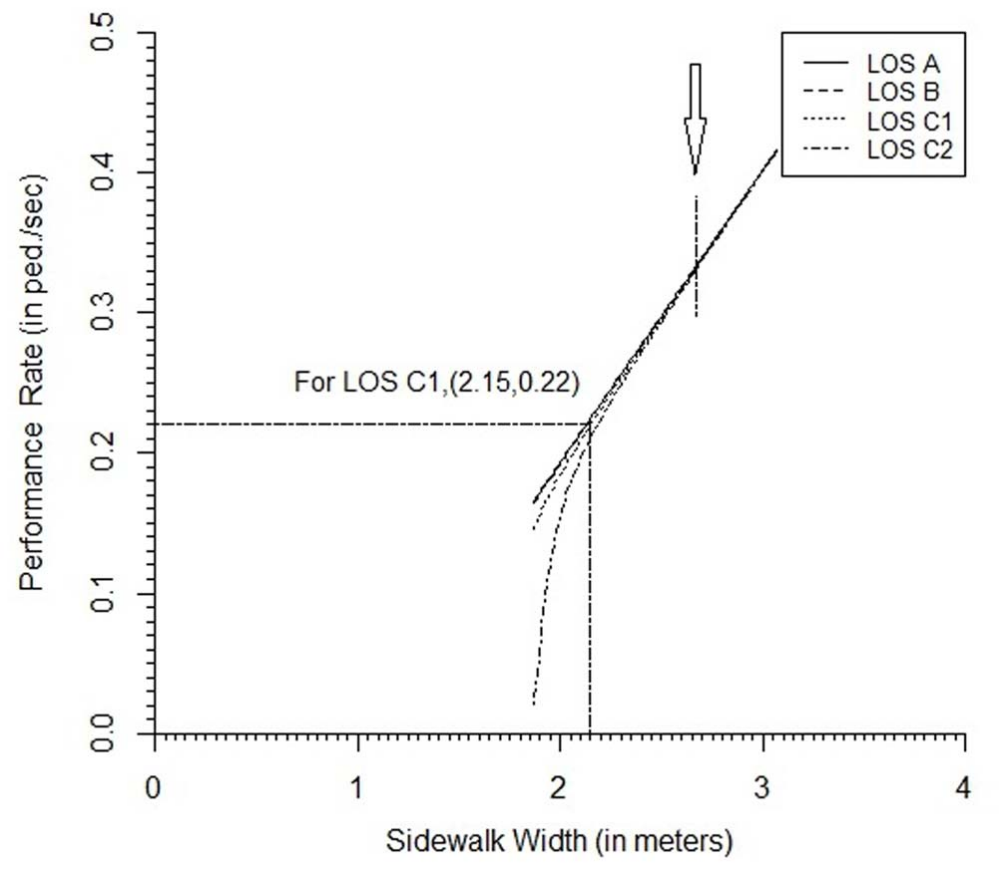
Effect of level of services and sidewalk width on performance.

Fig. 4 shows the predicted performances or overall service rates versus sidewalk width for different level of services of A, B, C_1_ and C_2_ (that is, one prevailing density for each LOS, that is, k = 0.30, 0.68, 1.20 and 1.5, respectively) as defined by Plus et al. [12]. Each of the four curves represents a particular LOS. It is obvious that as the sidewalk width increases, the overall service rate or performance also increases. The relationship between the sidewalk width and the performance rate is not in unique form. It is seen that the relationship is non-linear up to the sidewalk width of 2.67 metres. That width seems to be the effective width for the selected sidewalk. However, the relationship for the width past the effective width is exactly linear for different prevailing densities or LOS. In addition, it should be noted that the other parameters: free flow speed and maximum observed density will also have influence on the relationship between the performance rate and the sidewalk width. The gain of the developed model here reclines to some extent in the ability to predict the width-performance rate relationship more precisely for a particular LOS. Thus it can also provide an idea of efficient width for a walkway at a given observed maximum density and the free flow speed ability of pedestrians.

Fig. 4 also shows that, when the width is less than the effective width, the increment in overall service rate is not the same, and is dependent on the prevailing density or required LOS. For example, from the figure it is clear that if a traffic engineer in Dhaka has to pass 800 peds/hour (i.e.,0.22 ped/sec) through a sidewalk like the Commissioner’s Market Sidewalk, Farmgate at the average prevailing density 1.2 peds/m^2^ or LOS C_1_, the designer would have to use a 2.15-m width for the sidewalk. In other words, to evacuate a public gathering (e.g., a political party public meeting), which should follow the walking conditions on sidewalks in Dhaka at 800 peds/hour and the density of 1.2 peds/m^2^ and if there is only one exit available of 2.15-m width, the crowd needs to be controlled the exit point. At the control point on an average 22 pedestrians should be allowed to exit in each interval of 10 sec.

**Figure 5:**
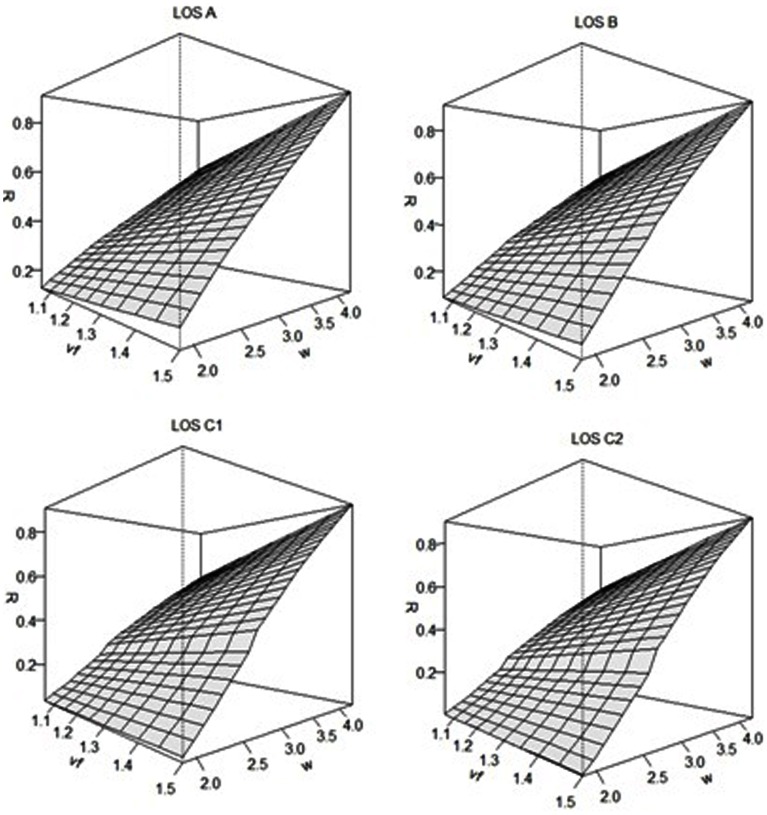
Relationships of free flow speed and sidewalk width with the performance for different level of services.

In addition, Fig. 4 also shows that, when the width is less than the effective width, as the prevailing density increases or LOS decreases, the overall service rate decreases. Obviously, the performance rate is lowest when the prevailing density is 1.5 peds/m^2^ of LOS C_1_. One can thus correlate the (overall service rate) performance to the LOS as mentioned in the first and third sections. For different level of services, the estimated performances are illustrated graphically in Fig. 5 at different combination of the values of free flow speed and sidewalk width.

From Fig. 5 it is clear that at higher level of service, pedestrians with higher capacity of free flow speed can accelerate the performance of a facility. However, when the prevailing LOS decreases this acceleration is not more effective. It is because as the LOS decreases the faster pedestrian movement is hindered by the forwarded pedestrians and thus the overall service rate cannot be expedited by the pedestrians with higher capacity of free flow speed. It is also clear from Fig. 5 that the performance rate increases gradually with the width of the sidewalk at higher LOS; whereas this rate increases rapidly up to the width of 2.67 metres (the width required to move two pedestrians concurrently) and then increases gradually with the width at lower LOS. In addition, the LOS decreases with the ranges of performance rate corresponding to each type of pedestrians (slower and faster). The variation in the ranges is bigger for faster pedestrians as compared to slower pedestrians.

### Conclusions and Limitations

In the limitation of comprehensible guidelines that incorporate the relevant factors and functions, the design and analysis of pedestrian facilities cannot be efficiently done. The efficient pedestrian facility is the one that can provide an adequate LOS to the pedestrians. The LOS is a qualitative evaluation of traffic flow characteristics and can be correlated to the performance of facility. Cost-effective pedestrian facilities thus result in optimal land use and ensuring prompt services to the pedestrians. The main objective of the current study, therefore, is to develop a model to assess the performance of a pedestrian facility in terms of LOS, so that the traffic engineers concerned can understand how well a particular sidewalk makes comfort and convenient pedestrian movements possible. The model also confirms the optimal land use for the pedestrian facilities. In this paper, a queuing based model is developed that is a new endeavour to model the pedestrian travel time. The developed model can be used to assess the overall service rate or performance of a facility and thus provide a guide to the designer of a pedestrian facility to determine the cost-effective sizing of the facility.

A better understanding of the pedestrian flow is possible to gain by the current developed model than the earlier models. One of the potential advantages of the model developed here is the ability to assess the influence of the relevant determinants and functional factors on the design and improvement of a pedestrian facility. The model is also able to test the plan of a pedestrian facility prior to construction to identify whether the facility would provide the expected services to pass a targeted number of pedestrians in a certain interval of time. In addition, the model could be helpful to control the pedestrian flows to meet certain performance rate. This is necessary as the effect of one pedestrian trying to walk faster than the pedestrians around her/him in a congested traffic is to force an opposing lane of pedestrians to split in two, which in turn reduces total flow.

It is important to note that although the use of queuing based travel time model explicitly facilitated the incorporation of important factors in the designing and planning of pedestrian facilities, the adequate space required for the supplementary activities (e.g. space for vendors) is not accounted for directly. A proper modification of the developed model may be useful for this purpose. In the development of the model, it is assumed that the arrival process follows a Poisson process and the inter-arrival times and the service time are exponentially distributed. This assumption may not always be suitable and any general distribution could be followed by the inter-arrival times and the service. Another limitation of the model is that it is a stationary approach, yet the non-stationary or transient nature is a common phenomenon. Thus a further effort is necessary to model the non-stationary environments.

Finally, this study focussed on instance of travel time/density, yet another study can be done based on the travel time/flow relationship. The pedestrian flow is actually a function of the LOS. In general, the pedestrian flow influences and also in turn, is influenced by the LOS. Such consideration can be utilized to develop a more superior model for the design and analysis of pedestrian facilities.

## Supporting Information

Appendix S1 **The Required Codes for the Sensitivity Analysis.**
(DOCX)
