# Supplementary material for: Modelling Pedestrian Travel Time and the Design of Facilities: A Queuing Approach
Source: PLoS One. 2013 May 15;8(5):e63503. doi: 10.1371/journal.pone.0063503 (PMC3655143; doi:10.1371/journal.pone.0063503)
Supplement: Appendix S1 — The Required Codes for the Sensitivity Analysis. (DOCX) [file pone.0063503.s001.docx]

**Appendix S1** The Required Codes for the Sensitivity Analysis

# The following codes were used in 2D Sensitivity Analysis

Reval<-function(s,vf,k)

{

L<-6.6

kj<-1.55

s1<-s-1

n<-length(s)

xsum<-0

for(j in 1:n){s1j<-s1[j];for(i in 0:s1j){xsum<-xsum+(k/kj)^i/factorial(i)}}

denom<-s*(1-k/(s*kj))*(k/kj)^s+s*factorial(s)*(1-k/(s*kj))^2*xsum

w<-1.07+0.8*s

Reval<-s*vf/(L*(1+(k/kj)^s/denom))

cbind(w,Reval)

}

rs1<-Reval(c(seq(1,2,.01),seq(2,2.753,.5)),1.1,.3)

rs2<-Reval(c(seq(1,2,.01),seq(2,2.753,.5)),1.1,.68)

rs3<-Reval(c(seq(1,2,.01),seq(2,2.753,.5)),1.1,1.2)

rs4<-Reval(c(seq(1,2,.01),seq(2,2.753,.5)),1.1,1.5)

rs<-data.frame(rs1[,1],rs1[,2],rs2[,2],rs3[,2],rs4[,2])

plot(rs1,type="n",frame=F,xlab="Sidewalk Width (in meters)",

ylab="Performance Rate (in ped./sec)",

xlim=c(0,4),ylim=c(0,.5),xaxs="i",yaxs="i")

axis(side=1,at=seq(0,4,by=0.05),labels=F,tcl=-.2)

axis(side=2,at=seq(0,.5,by=0.01),labels=F,tcl=-.2)

lines(rs1,lty=1)

lines(rs2,lty=2)

lines(rs3,lty=3)

lines(rs4,lty=4)

legend("topright",c("LOS A","LOS B","LOS C1","LOS C2"),

cex=0.8,lty=1:5)

#abline(h=.22,lty=3)

#coords <- locator(type="l")

#coords

#abline(v=coords$x,lty=3)

text(1.4,.25,"For LOS C1,(2.15,0.22)",cex=.9)

lines(cbind(c(0,2.15),c(.22,.22)),lty=6)

lines(cbind(c(2.15,2.15),c(0,.22)),lty=6)

lines(cbind(c(2.67,2.67),c(0,.5)),lty=6)

# The following codes were used in 3D Sensitivity Analysis

Reval<-function(s,vf,k)

{

L<-6.6

kj<-1.55

s1<-s-1

n<-length(s)

xsum<-0

for(j in 1:n){s1j<-s1[j];for(i in 0:s1j){xsum<-xsum+(k/kj)^i/factorial(i)}}

denom<-s*(1-k/(s*kj))*(k/kj)^s+s*factorial(s)*(1-k/(s*kj))^2*xsum

w<-1.07+0.8*s

Reval<-s*vf/(L*(1+(k/kj)^s/denom))

}

vf<-seq(1.05,1.5,.05)

w<-seq(1.87,4.27,.1)

R<-rep(0,length(vf)*length(w))

dim(R)<-c(length(vf),length(w))

rownames(R)<-vf

colnames(R)<-w

for(i in 1:length(vf))

{for(j in 1:length(w))

{R[i,j]<-Reval((w[j]-1.07)/.8,vf[i],1.5)

}}

#For different value of k, we will have differnt 3D graphs. Here, k=1.5 or LOS C2

persp(x=vf,y=w,z=R,expand=.9,theta=48,phi=00,shade=.15,

ltheta=60,ticktype="detailed")

mtext("LOS C2")

forgap<-round(R,2)

#To identify the jump in 3D graph we can use the codes in the above line.
